# An Elbow Patch Reconstruction Technique for Narrowed Remnant Portal Veins during Right Lobe Living Donor Hepatectomy: A Rescue Surgery

**DOI:** 10.3390/jcm13102924

**Published:** 2024-05-16

**Authors:** Sertac Usta, Sami Akbulut, Kemal Baris Sarici, Ibrahim Umar Garzali, Fatih Ozdemir, Fatih Gonultas, Adil Baskiran, Burak Isik, Sezai Yilmaz

**Affiliations:** 1 Department of Surgery and Liver Transplant Institute, Inonu University Faculty of Medicine, Malatya 44280, Turkey; sertacusta44@gmail.com (S.U.); fatihup@hotmail.com (F.O.); fatnih44@gmail.com (F.G.); dr.adil.baskiran@gmail.com (A.B.); isik_burak@yahoo.com (B.I.); sezai.yilmaz@inonu.edu.tr (S.Y.); 2 Department of Surgery, Aminu Kano Teaching Hospital, Kano 700101, Nigeria

**Keywords:** living donor liver transplantation, living donor hepatectomy, portal vein narrowing, elbow patch, homologous vascular grafts

## Abstract

**Background:** Treatment of established portal vein narrowing after living donor hepatectomy is challenging. We aimed to present a new approach termed the “elbow patch reconstruction technique” to correct the narrowed remnant portal vein just or late after right lobe living donor hepatectomy. **Methods:** Demographic and clinical data of 12 living liver donors with narrowed remnant portal veins and treated with the “elbow patch reconstruction technique” were prospectively collected and retrospectively evaluated. Anatomic variation of the portal vein was defined in accordance with the Nakamura classification; six of the living liver donors had type A, three had type B, and the remaining three had type C. In eight of the living liver donors with a narrowed remnant portal vein, diagnosis was detected by intraoperative Doppler ultrasonography and visual inspection by experienced transplant surgeons in the living donor hepatectomy procedure. In the remaining four living liver donors, diagnosis was performed postoperatively when elevation of liver enzymes was noticed during the routine liver function test and Doppler US. The diagnosis was confirmed by multidetector computed tomography. **Results:** Data from nine males and three females aged 18 to 54 years were analyzed. All of the living liver donors were followed up for a median of 1710 days (min-max: 1178–4447 days; IQR: 1516 days), and none of the living liver donors had any structural or functional complications in the portal vein. **Conclusions:** Narrowing remnant portal veins are rare, but they are a life-threatening complication in living liver donors, and this condition requires urgent management. Image guided interventions and narrowed segment resection with end-to-end anastomosis using a vascular graft carried a potential risk for thrombosis and restenosis. To avoid these complications, we shared a technique named “elbow patch reconstruction technique”. This technique can be very effective in relieving the narrowing of the remnant portal vein after right lobe living donor hepatectomy.

## 1. Introduction

The concept of living donor liver transplantation (LDLT) was proposed by Smith and colleagues [1] in 1969, but it was not attempted until December 1988, when Raia and colleagues [2] attempted the first LDLT on a 4-year-old boy who died 6 days after the transplant. In July 1989, the first successful LDLT was performed by Strong and colleagues [3] when the left lateral segment (segments II and III) was transferred from the mother into her child. LDLT became accepted in children in a few years, and helped to reduce the mortality of patients on the waiting list. Right lobectomy for adult-to-adult LDLT was more complex, and it was performed for the first time by Yamaoka and colleagues [4] in Japan in 1994.

At the initial stage, there was strong skepticism about LDLT, especially the use of right lobe grafts for adult recipients [5,6,7,8]. Most of the skeptics believed the act of subjecting a healthy living donor to a major procedure was against one of the fundamental ethics of medical practice: “primum non nocere” (first do no harm) [6,7,8]. However, with refinement of surgical skills and updates in living liver donor evaluation, the transplant community has accepted that the benefit of transplantation to an appropriately selected recipient is worth subjecting an appropriately selected living liver donor to a minimal risk.

Living donor hepatectomy was associated with significant morbidity rates in living liver donors in the early 1990s [9]. There was a significant improvement in living liver donors’ morbidity over the years as transplant surgeons became more proficient in preoperative evaluation, intraoperative techniques, and postoperative management [10]. Initial studies reported living liver donor complications of up to 50%, but over the decades, living liver donor complications have been reported at around 10–15% [9]. Biliary complications are considered the most common morbidity experienced by living liver donors and can occur in up to 9% of living liver donors [11,12,13,14,15].

**Figure 1:**
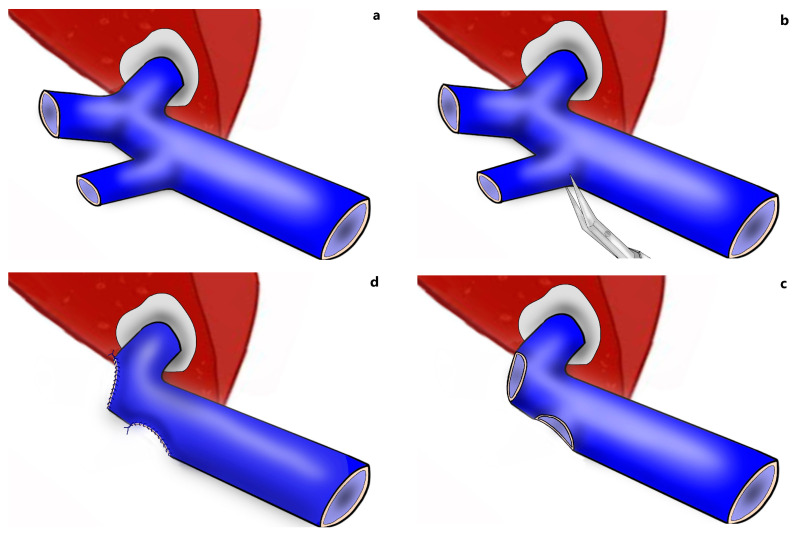
Portal vein configurations. (**a**) Anomalous portal venous branching in the right lobe; (**b**,**c**) cutting the portal vein branches of the right lobe; (**d**) narrowed portal vein after closure of stump of the right portal vein branches.

While biliary complications are considered the most common after living donor hepatectomy [15], portal vein complications are rare, occurring in 0.1–3.8% of patients [16,17,18]. Although portal vein complications after living donor hepatectomy are rare, it should be considered that these complications are associated with significant morbidity and even mortality in patients [19]. Narrowing of the remnant portal vein at the take-off point of the right portal vein after right lobe living donor hepatectomy is a rare, but encountered, complication. The anatomy of the portal bifurcation is such that the left branch takes off at a sharp angle, whereas the course of the right branch is almost in line with the main portal trunk. The donor surgeon’s effort to remove a long right portal vein with a right lobe graft by encroaching on the borders of the main portal vein will inevitably cause stenosis and subsequent thrombus in the portal vein. Especially in the presence of anomalous portal venous branching in the right lobe (Figure 1a), the donor surgeon’s effort to remove the right portal vein as a single lumen is the most common cause of portal vein thrombosis after living donor hepatectomy [20,21,22]. In other words, at the immediate post-operative period, narrowing of the remnant portal vein may precipitate portal vein thrombosis, and this may become a life-threatening complication by causing portal hypertension, mesenteric congestion, and sepsis, which increase postoperative morbidity and mortality [17,21]. Long-term stenosis is associated with portal hypertension and its complications, which may be a significant source of morbidity for some donors after right lobe living donor hepatectomy [12,19,20,23,24].

There have been attempts by some surgeons to prevent this narrowing while obtaining the maximum length of the right portal vein during right lobe living donor hepatectomy. Marcos and colleagues [20] described the use of intraoperative Doppler ultrasound after clamping the right portal vein to ensure adequate diameter and flow within the main portal and the remnant left portal vein. If the flow and diameter were diminished, they recommended moving the clamp towards the right lobe liver until an adequate flow and diameter was achieved. Another technique described is the vertical application of the portal vein clamp utilized by Yanaga and colleagues [24] to reduce the risk of narrowing of the remnant portal vein.

Treatment of established portal vein narrowing post living donor hepatectomy is challenging. Surgical resection of the narrowed segment with replacement with either cryopreserved homologous or prosthetic has been proposed with good results [25,26]. However, this may be associated with restenosis, especially at the anastomotic site, which will subsequently cause portal hypertension and its sequelae. The role of postoperative portal venous stent placement has also been discussed [27,28,29]. The approach for stent placement can be transhepatic, percutaneous, or through the ileocolic vein. The use of stents has been associated with some complications like stent migration, thrombosis, sepsis, and occlusion [27,28,29]. In cases diagnosed after the operation, a stent can be placed in the narrow segment of the portal vein by interventional radiologists [30]. In cases of portal vein stenosis or thrombosis after living donor hepatectomy, this problem must be resolved during the operation, and reconstruction should not be avoided even in cases of suspected stenosis. We aimed to present a new approach, termed the “elbow patch reconstruction technique”, to correct the narrowed remnant portal vein just or late after right lobe living donor hepatectomy.

## 2. Materials and Methods

### 2.1. Description of the Study and Parameters

Between September 2005 and January 2022, a total of 1875 living donor hepatectomy procedures were performed in our liver transplantation institute. Demographic and clinical data of 12 living liver donors with narrowed remnant portal veins were prospectively collected and retrospectively evaluated. Anatomic variation in the portal vein was defined according to the Nakamura classification [31]: Type A: bifurcation of the right portal vein and left portal vein; Type B: trifurcation of the right anterior portal vein, right posterior portal vein, and left portal vein; Type C: extraparenchymal branching of the right anterior portal vein from the left portal vein; Type D: intraparenchymal branching of the right anterior portal vein from the left portal vein; and Type E: branches of segments VIII and V originate separately from the left portal vein. The classification of the portal vein variations is shown with details in Table 1 [31]. Anatomic variation in hepativc arterial system was defined according to Michel’s classification [32]: type I: normal anatomy; type II: replaced left hepatic artery from the left gastric artery; type III: replaced right hepatic artery from the superior mesenteric artery; type IV: replaced right hepatic artery and left hepatic artery; type V: accessory left hepatic artery; type VI: accessory right hepatic artery; type VII: accessory right hepatic artery and left hepatic artery; type VIII: a replaced right hepatic artery or left hepatic artery with other hepatic artery being an accessory one; type IX: the hepatic trunk as a branch of the superior mesentericartery; and type X: the common hepatic artery from the left gastric artery. Anatomical variations in the intrahepatic bile ducts were defined according to Choi classification [33]: Type 1: typical feature; Type 2: simultaneous opening of the right anterior hepatic duct, right posterior hepatic duct and left hepatic duct into the common hepatic duct; Type 3: right posterior segmental duct drains anomalously (3A: drains into the LHD; 3B: into the common hepatic duct; 3C: into the cystic duct); Type 4: right hepatic duct drains into the cystic duct; Type 5: an accessory duct is present (5A: into the common hepatic duct; 5B: into the right hepatic duct); Type 6: segments II and III drain individually into the right hepatic duct or common hepatic duct; and Type 7: unclassified or complex variation.

In eight of the living liver donors with a narrowed remnant portal vein, diagnosis was achieved by intraoperative Doppler ultrasonography and visual inspection by experienced transplant surgeons. In the remaining four living liver donors, diagnosis was performed postoperatively when elevation of liver enzymes was noticed during the routine liver function test and Doppler ultrasonography after living donor hepatectomy procedures. The diagnosis was confirmed by multidetector computed tomography. Three of the twelve living liver donors in this study had previously been presented in two different studies, but technical details were not provided in these presentations [21,22]. Intraoperative and postoperative surgical complications were categorized based on the “Clavien Surgical Morbidity Scale Modified for Living Donors” scale, developed by Barr et al. [34] at the Vancouver Forum in 2005 and later modified by Cheah et al. [35] in 2013. Since the INR value was slightly above normal limits in the early postoperative period, living liver donors were not given any anticoagulant therapy after the elbow patch reconstruction procedure.

### 2.2. Elbow Patch Reconstruction Technique

**Figure 2:**
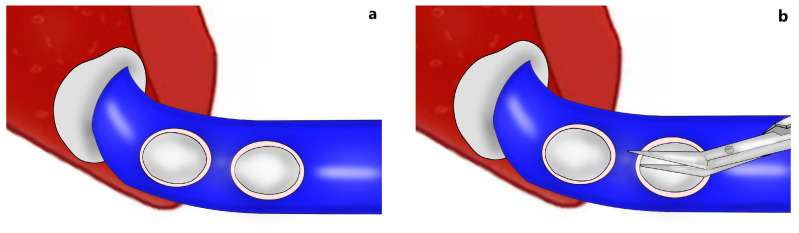

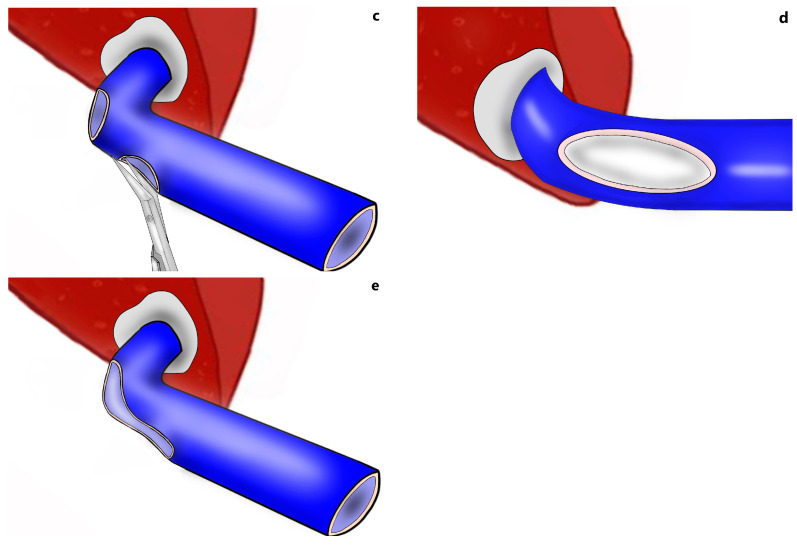
Preparation of the narrowed segment for reconstruction: (**a**) the sutures in both portal venous stumps leading to the right lobe are removed; (**b**,**c**) the venous bridge between both lumens was cut (front and side view); (**d**,**e**) turning into one lumen (front and side view).

**Figure 3:**
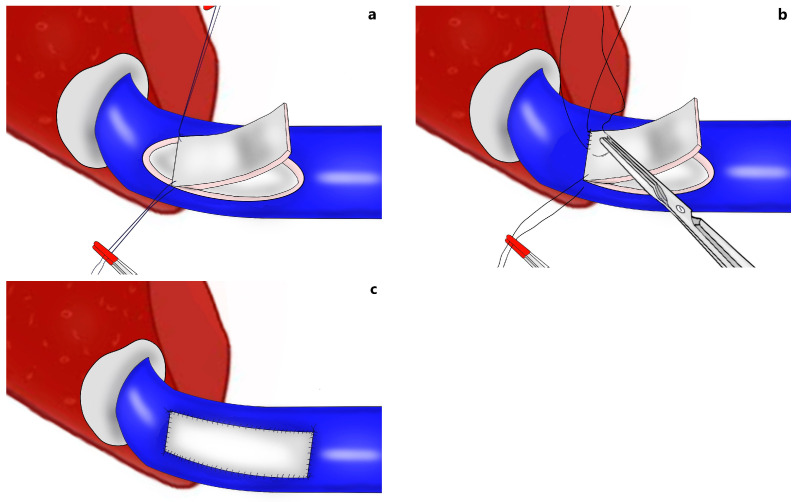
Reconstruction of narrowed portal vein by venous patch: (**a**) two corner sutures are placed 0.5 cm inferior of the upper pole of the oval opening to turn into a rectangular shape; (**b**,**c**) all-round suturing of the venous patch is completed.

**Figure 4:**
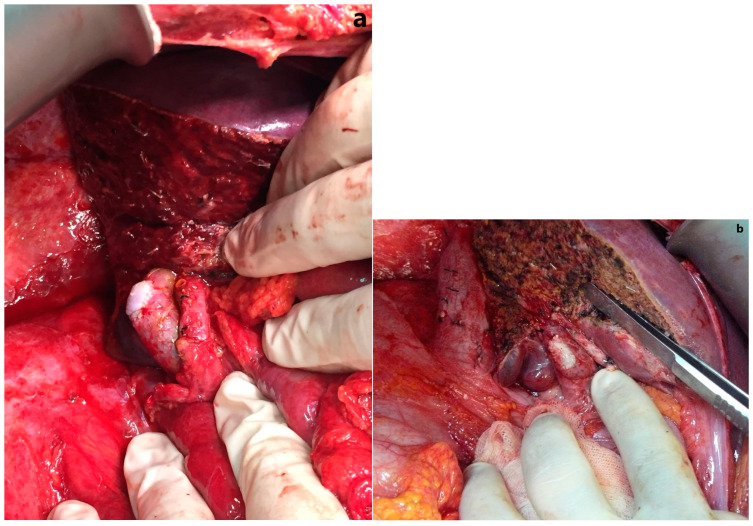
Intraoperative view of the repaired portal vein: (**a**) front view; (**b**) right side view.

When a narrowed remnant portal vein segment is detected following closure of the right portal vein stump (Figure 1b–d), vascular clamps are placed both proximal and distal to the narrowed portal vein segment. Then, the sutures in the narrowed portal vein segment were removed. In type C anomalous portal venous branching cases, the sutures in both portal venous stumps leading to the right lobe were removed (Figure 2a). The venous bridge between both lumens was cut (Figure 2b,c) and turned into one lumen (Figure 2d,e). When viewed from the front and right sides, the venous opening generally appears as a longitudinal oval shape along the portal vein axis. Two corner sutures are placed 0.5 cm inferior of the upper pole of this oval structure (Figure 3a). The oval shape has now turned into a rectangular shape. The sutures used are 7/0 or 8/0 polypropylene. After checking endothelial integrity, a cryopreserved patchy venous graft was cut to the appropriate size on the back-table. At this stage, we usually cut the venous patch in accordance with the upper corners and start suturing the parts between the corner sutures at the most difficult point. When approaching the inferior poles, the venous patch is trimmed again to suit the portal venous opening. All-round suturing of the venous patch is completed (Figure 3b). It is confirmed that the venous patch applied to the portal venous elbow by removing portal venous clamps widens the stenosis satisfactorily (Figure 4a,b). After this stage, one more check is made with intraoperative Doppler ultrasonography.

### 2.3. Study Protocol and Ethics Committee Approval

This study involving human participants was conducted according to the ethical standards of the institutional and national research committee and the 1964 Helsinki Declaration and its later amendments or comparable ethical standards. Ethical approval was obtained from the Inonu University Institutional Review Board (IRB) for non-interventional studies (Approval No: 2019/9-26). The Strengthening the Reporting of Observational studies in Epidemiology (STROBE) guideline was utilized to assess the likelihood of bias and overall quality for this study [36].

## 3. Results

**Figure 5:**
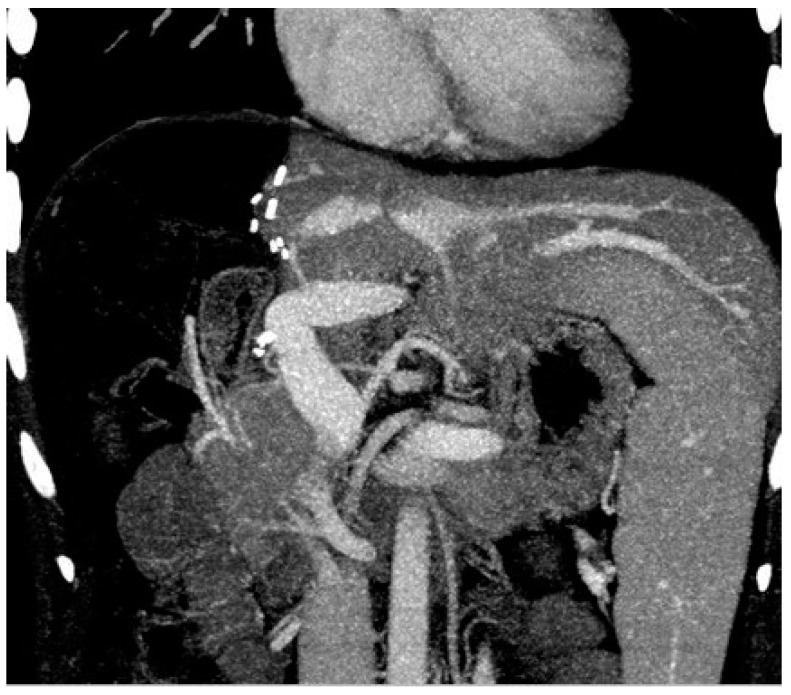
The coronal reformatted computed tomography imaging shows that the portal vein reconstructed with the elbow patch technique is patent.

Data of nine males and three females aged 18 to 54 years were analyzed. Six of the living liver donors had Nakamura type A, three had Nakamura type B, and the remaining three had Nakamura type C. All Type B and C cases had two separate stumps, but there was also remnant portal vein stenosis. While the narrowed remnant portal vein in eight of the living liver donors was detected during the living donor hepatectomy procedure, narrowing was detected in four of the living liver donors during postoperative radiological investigations. All living liver donors were followed up for a median of 1710 days (min–max: 1178–4447 days; IQR: 1516 days), and none of the living liver donors had any structural or functional complications in the remnant portal vein (Figure 5). Some demographic and clinical characteristics are summarized in Table 2.

## 4. Discussion

LDLT faced initial skepticism because of the fear of living liver donors’ morbidity to a supposedly healthy person undergoing a major surgery like right lobe living donor hepatectomy [5,6,8,37]. To reduce this morbidity, extensive preoperative evaluation of living liver donors has been integrated into the practice of LDLT [38]. The preoperative evaluation is aimed at a systemic evaluation to ensure that the living liver donor has no systemic changes that will precipitate morbidity or mortality during or after surgery. An equally important component of living liver donor evaluation is the assessment of the architecture, function, and vasculature of the liver. The architectural assessment is aimed at identifying steatosis or fibrosis in a donor, as this may precipitate post-operative hepatic failure in living liver donors. Another component of hepatic assessment is the vasculature and drainage [20,39]. The supposedly normal anatomy of hepatic vasculature and drainage is seen in just over 50% of normal individuals, so anatomic abnormalities should be expected and identified during donor evaluation since the inability to properly identify these abnormalities may precipitate significant morbidity in living liver donors after LDLT [40].

The anatomic configuration of the portal vein is such that the left portal vein takes off from the main portal vein at an angle, while the right portal vein continues in almost the same axis as the main portal vein. This configuration predisposes living liver donors undergoing right lobe living donor hepatectomy to narrowing or angulation in the right portal vein stump between the remnant left portal vein and the main portal vein. Another cause of the narrowing of the remnant portal vein during right lobe living donor hepatectomy is seen when the patients have anomalous portal venous branching in the right liver lobe. In Type B cases, the donor surgeon’s attempts to obtain a single-lumen portal vein in the right lobe graft will cause stenosis in the main and left portal vein axes leading to the remnant left lobe. In Type C cases, when two separate right portal vein stumps are closed, it may cause stenosis in the portal vein due to the plane difference in the suture lines. The narrowing may predispose to portal vein thrombosis just or at the immediate post-operative period, and this may result in acute portal hypertension, mesenteric congestion, sepsis and post-operative liver failure, all of which increase postoperative morbidity and mortality. If the narrowing does not cause thrombosis in the immediate postoperative period, patients will develop long term portal hypertension from the stenosis, and this will result in ascites and variceal bleeding.

Many methods have been proposed to prevent portal vein stenosis after right lobe living donor hepatectomy. Marcos and colleagues [20] proposed the use of intraoperative Doppler ultrasonography to identify the appropriate length of the right portal vein before transection. What they proposed was the initial application of a portal clamp to the point of origin of the right portal vein, followed by Doppler ultrasonography to assess the diameter and flow through the remnant portal vein. If the diameter or flow is reduced, then the clamp is adjusted towards the liver until adequate flow and diameter are established before the right portal vein is transected. Another way of preventing the narrowing was the vertical clamping proposed by Yanaga and colleagues [24]. In this technique, they proposed the application of a clamp to the right portal vein along the long axis of the main portal vein in alignment with the right edge of the main portal vein. They noticed that this technique of clamping does not cause narrowing or angulation of the remnant portal vein.

Treatment of the narrowed remnant portal vein after right lobe living donor hepatectomy is important to prevent the complications mentioned. Surgical excision of the narrowed segment with replacement and anastomosis of the proximal and distal segments was first described by Scantlebury and colleagues [26]. However, this may be associated with restenosis, especially at the anastomotic site, which will subsequently cause portal hypertension and its sequelae. If the two edges of the vein cannot come together, the resected portion of the vein can be replaced with a cryopreserved homologous/autologous vascular graft or prosthetic graft [22,25,26,27,28,41,42]. The use of a vascular or prosthetic graft may be associated with thrombosis. Another option is the use of tubularized autologous peritoneal patch, as described by Sabuncuoglu and colleagues [42]. For cases of narrowing discovered after surgery, the role of stent placement has been discussed [27,28,29]. The approach for stent placement can be percutaneous transhepatic or through the ileocolic vein. The use of stents has been associated with some complications like stent migration, thrombosis, sepsis, and occlusion [27,28,29].

We propose a novel technique for correcting portal vein narrowing during right lobe living donor hepatectomy. We presented the first trials of this technique in two different case studies before [21,22], and we continued to use the same approach, when necessary, based on the experiences we gained from those cases. However, we have not given any particular name to this technique before. The elbow patch technique was utilized for 12 patients with narrowing of the remnant portal vein after right lobe living donor hepatectomy with good results. The median follow-up of the patient is 1710 days, with no evidence of portal vein thrombosis, stenosis, or portal hypertension. The risk of re-stenosis with our technique is low, as more than half of the circumference of the vein is native to the vein, so there is no fibrosis at that point. In our study, we used cryopreserved homologous vascular grafts for the elbow patch technique. Other options to use for the patch include the use of peritoneum [42], a prosthetic material like polyethylene terephthalate (Dacron), or a prosthetic vascular graft such as expanded polytetrafluoroethylene (ePTFE) [41]. The use of peritoneum may have an advantage over the use of prosthetic material because it is an autologous tissue with less thrombogenic potential. It is also easier to handle, more flexible, and has an abundant supply in the abdomen.

## 5. Conclusions

As a conclusion, narrowing the remnant portal vein is a rare, but life-threatening complication in living liver donors, and this condition requires urgent management. Image guided interventions and narrowed segment resection with end-to-end anastomosis using a vascular graft carried a potential risk for thrombosis and restenosis. To avoid these complications, we shared a technique named “elbow patch” reconstruction technique. This technique can be very effective in relieving the narrowing of the remnant portal vein after right lobe living donor hepatectomy.

## Figures and Tables

**Table 1:** Classification of the portal vein branching according to the schema proposed by Nakamura et al. [31].

Type	Definitions of Nakamura Classifications	View
Type A	Bifurcation of the right portal vein and left portal vein (usual bifurcation type)	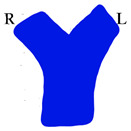
Type B	Trifurcation of the right anterior portal vein, right posterior portal vein, and left portal vein	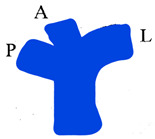
Type C	Extraparenchymal branching of the right anterior portal vein from the left portal vein	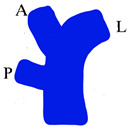
Type D	Intraparenchymal branching of the right anterior portal vein from the left portal vein	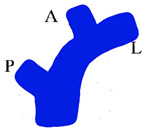
Type E	Branches of segments 5 and 8 originate separately from the left portal vein	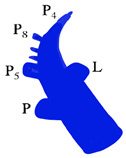

**Table 2:** Demographic and clinical features of 12 living liver donors with narrowed portal veins.

Sex	Age	BMI	Relationship to Recipient	PV Type (Nakamura) [31]	Bile Ducts (Choi) [33]	Artery Type (Michaels’) [32]	Liver Graft (gram)	Follow Up (Days)
M	54	27	Brother	Type-A	Two orifices	Type-I	870	4414
F	31	NA	Daughter	Type-A	Two orifices	Type-II	650	3440
M	20	NA	Son	Type-C	Two orifices	Type-II	971	2979
F	22	26	Daughter	Type-C	Two orifices	Type-V	500	2696
M	32	26	Son	Type-B	Two orifices	Type-V	780	2283
F	21	20	Daughter	Type-A	Type-IIIB	Type-II	650	1750
M	34	23	Son	Type-A	Type-II	Type-I	675	1603
M	27	29	Son	Type-A	Type-I	Type-II	1060	1508
M	26	24	Cousin	Type-A	Two orifices	Type-I	875	1351
M	18	25	Son	Type-B	Type-IIIA	Type-I	800	1292
M	33	25	Son	Type-B	Two orifices	Type-II	756	1285
M	23	16	Brother-in-law	Type-C	Type-IIIA	Type-V	610	1145

## Data Availability

The datasets analyzed during the current study are available from the corresponding author on reasonable request.
